# Gene expression analysis reveals marked differences in the transcriptome of infantile hemangioma endothelial cells compared to normal dermal microvascular endothelial cells

**DOI:** 10.1186/2045-824X-5-6

**Published:** 2013-03-25

**Authors:** Jessica M Stiles, Rebecca K Rowntree, Clarissa Amaya, Dolores Diaz, Victor Kokta, Dianne C Mitchell, Brad A Bryan

**Affiliations:** 1 Department of Biomedical Sciences, Paul L. Foster School of Medicine, Texas Tech University Health Sciences Center, El Paso, TX, USA; 2 Department of Pathology, CHU Sainte-Justine, University of Montreal, Montreal, QC, Canada

## Abstract

**Background:**

Infantile hemangiomas are benign vascular tumors primarily found on the skin in 10% of the pediatric population. The etiology of this disease is largely unknown and while large scale genomic studies have examined the transcriptomes of infantile hemangioma tumors as a whole, no study to date has compared the global gene expression profiles of pure infantile hemangioma endothelial cells (HEMECs) to that of normal human dermal microvascular endothelial cells (HDMVECs).

**Methods:**

To shed light on the molecular differences between these normal and aberrant dermal endothelial cell types, we performed whole genome microarray analysis on purified cultures of HEMECs and HDMVECs. We then utilized qPCR and immunohistochemistry to confirm our microarray results.

**Results:**

Our array analysis identified 125 genes whose expression was upregulated and 104 genes whose expression was downregulated by greater than two fold in HEMECs compared to HDMVECs. Bioinformatics analysis revealed three major classifications of gene functions that were altered in HEMECs including cell adhesion, cell cycle, and arachidonic acid production. Several of these genes have been reported to be critical regulators and/or mutated in cancer, vascular tumors, and vascular malformations. We confirmed the expression of a subset of these differentially expressed genes (ANGPT2, ANTXR1, SMARCE1, RGS5, CTAG2, LTBP2, CLDN11, and KISS1) using qPCR and utilized immunohistochemistry on a panel of paraffin embedded infantile hemangioma tumor tissues to demonstrate that the cancer/testis antigen CTAG2 is highly abundant in vessel-dense proliferating infantile hemangiomas and with significantly reduced levels during tumor involution as vascular density decreases.

**Conclusion:**

Our data reveal that the transcriptome of HEMECs is reflective of a pro-proliferative cell type with altered adhesive characteristics. Moveover, HEMECs show altered expression of many genes that are important in the progression and prognosis of metastatic cancers.

## Introduction

Infantile hemangiomas are benign tumors of vascular origin that affect approximately 10% of the pediatric population. These tumors are characterized by a rapid proliferation phase over the first 1–2 years of the child’s life, followed by a slow and steady decline over the next 5–7 years leading to the complete involution of the tumor mass. Approximately 90% of all infantile hemangiomas remain small and are best left alone to naturally involute. However in about 10% of the cases the tumors exhibit aggressive characteristics based on their size, location, number, etc. and must be actively treated to avoid patient disfigurement and/or mortality.

The etiology of infantile hemangiomas is largely unknown, particularly with regard to the cellular origin of the tumor. Circumstantial evidence suggests that these lesions are of aberrant placental origin as evidenced by upregulated Glut1 expression [1], and some labs have ventured to hypothesize that they may be formed from metastatic invasion of placenta-derived chorangioma cells [2]. Indeed, transcriptional profiling of human placenta, infantile hemangioma, and eight normal and diseased vascularized tissues suggests that high transcriptome similarity is shared between placenta and hemangioma tissues, more so than any of the other tissues tested [3]. Global gene expression analysis of infantile hemangioma tumors has been previously performed by two labs. Ritter et al. [4] utilized microarray analysis on whole tumors and identified immune regulators and indoleamine 2,3 dioxygenase as key regulators of infantile hemangioma involution. Calicchio et al. [5] utilized laser capture microdissection and genome-wide transcriptional profiling of vessels from proliferating and involuting hemangiomas. The authors strongly associated proliferating hemangioma vessels with increased expression of genes involved in endothelial-pericyte interactions and neuronal/vascular patterning, and involuting hemangiomas with chronic inflammatory mediators and angiogenic inhibitors. Given the high density of tightly associated pericytes in infantile hemangiomas and the inevitable collateral capture of intraluminal white cells, fibroblasts, mast cells, and perivascular collagen with laser microdissection, these data represent changes from numerous cell types within the infantile hemangioma tumor, but are not reflective specifically of the aberrant endothelial cells which contribute to disease. While these genomics studies have provided great mechanistic insight into the etiology and progression of the disease, they have not addressed the unique differences between abnormal infantile hemangioma endothelial cells and the normal dermal endothelial cells that are resident in the surrounding skin area of the patient. Understanding these differences could identify targetable pathways that could be exploited to preferentially block hemangioma growth and spread, but spare normal endothelial cells.

To date, no direct whole genome comparison of pure cultures of human dermal microvascular endothelial cells (HDMVECs) and infantile hemangioma endothelial cells (HEMECs) has been reported. To address this, we performed whole genome microarray profiling of the gene expression alterations between low passage pure cultures of HEMECs and HDMVECs. We identified a number of transcriptional alterations that are likely to contribute to the aggressive phenotype of infantile hemangiomas and that could potentially be utilized in immunotherapy against particularly aggressive hemangiomas tumors.

## Materials and methods

### Cell culture and chemicals

The HEMEC cell line was previously isolated from a proliferating-phase infantile hemangioma specimen collected from a female infant and generously donated to us by Joyce Bischoff (Harvard Medical School) [6]. The primary culture of neonatal HDMVECs was purchased from ATCC. Both cell lines were cultured as previously reported [7]. For all experiments, cell lines were used at <5 passages.

### Proliferation assay

Cells were plated at equivalent sub-confluent densities and maintained in a Nikon Biostation CT time lapse imaging station. Cell proliferation was measured by counting cells per vision field from 5 independent areas over a 96 hour time course. Data presented is the average of the counts plus or minus the standard deviation. Student’s *t*-test was used to evaluate statistical significance. Data with p<0.05 was considered significant.

### Migration assay

Confluent cultures were scratch wounded and the progress of “wound healing” was monitored using a Nikon Biostation CT time lapse imaging station over a 9 hour period. Data presented is the average migration speed plus or minus the standard deviation. Student’s *t*-test was used to evaluate statistical significance (p<0.05). Data with p<0.05 was considered significant.

### Immunofluorescence

Cells were plated onto collagen type I coated glass coverslips, fixed in 4% paraformaldehyde, and incubated with antibodies against phospho-focal adhesion kinase (p-FAK; 1:1000; Cell Signaling #3283), rhodamine conjugated phalloidin (1:350; Cytoskeleton Inc.), or DAPI and imaged via a Nikon Eclipse Ti laser scanning confocal microscope.

### Microarray analysis

Total RNA was amplified and biotin-labeled using Illumina TotalPrep RNA Amplification Kit (Ambion). 750 ng of biotinylated aRNA was then briefly heat-denatured and loaded onto expression arrays to hybridize overnight. Following hybridization, arrays were labeled with Cy3-streptavidin and imaged on the Illumina ISCAN. Intensity values were transferred to Agilent GeneSpring GX microarray analysis software and data was filtered based on quality of each call. Statistical relevance was determined using ANOVA with a Benjamini Hochberg FDR multiple testing correction (p-value < 0.05). Data were then limited by fold change analysis to statistically relevant data points demonstrating a 2-fold or more change in expression. Pathway analysis was performed using Metacore software. The microarray data from this experiment is publically available on the Gene Expression Omnibus (GEO Accession #GSE43742).

### Quantitative real time PCR analysis

RNA was isolated from cells using the Ambion Purelink Minikit according to the manufacturer’s directions. qRT-PCR was performed on an ABI7900HT RT-PCR system using TaqMan Assays with predesigned primer sets for the genes of interest (Invitrogen). All RT-PCR experiments were performed in triplicate.

### Immunohistochemistry

Paraffinized infantile hemangioma tissues were labeled with CTAG2 antibody (1:200, Santa Cruz Biotechnology #sc99243) and quantified using Alkaline Phosphatase detection (CellMarque). Positive and negative controls from breast carcinoma tissues were stained with CTAG2 antibody or sham, respectively. Use of de-identified human tissues was approved by the Texas Tech University Health Sciences Center Institutional Review Board for the Protection of Human Subjects (IRB E13029). Waiver of informed consent was approved by IRB.

## Results and discussion

**Figure 1:**
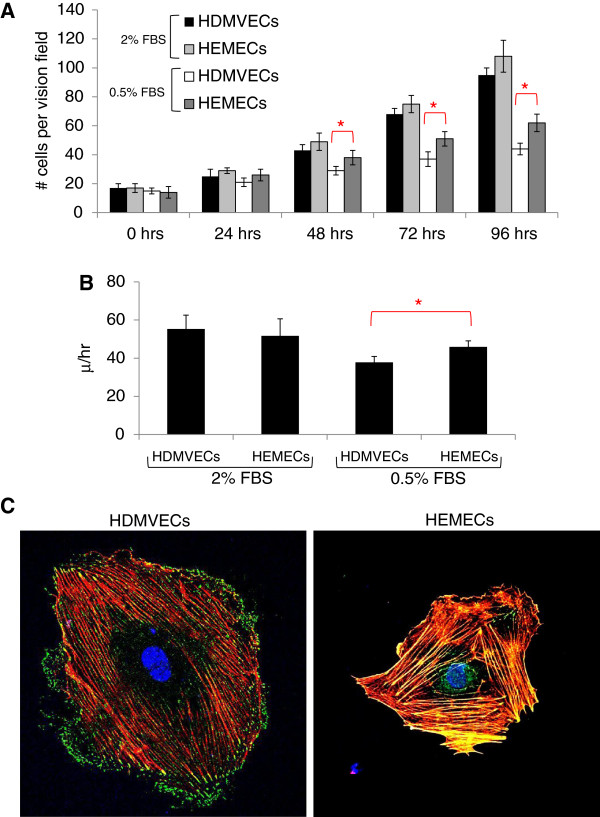
**Analysis of HDMVEC and HEMEC phenotypes.** (**A**) Analysis of proliferation rates between HDMVECs and HEMECs over a 48 hr time course. (**B**) Analysis of the migration rates of HDMVECs and HEMECs nine hours after initial scratch from a micropipette. (**C**) Immunofluorescent imaging of actin (*red*), p-FAK (*green*), and nucleus (*blue*). (red asterisks for panels **A** &**B** represent statistically significant values [p<0.05] as determined by Student’s *t*-test).

A comparison of the proliferation and migration rates of HEMECs and HDMVECs under standard growth conditions revealed no significant difference between normal and hemangioma endothelial cell types, however HEMECs grown under reduced serum conditions (0.5% fetal bovine serum) exhibited an approximately 30% increase in proliferation and an approximately 18% increase in migration relative to HDMVECs grown under the same conditions (Figure 1A & B). This suggests the higher serum concentrations were likely masking any phenotypic advantage attributed to the HEMECs. Moreover, it indicates the proliferative and migratory capacity of HEMECs are unique from that observed in HDMVECs and agrees with earlier reports suggesting advantages in these areas for HEMECs [6]. Comparisons of fluorescent images of the actin cytoskeleton and active focal adhesion complexes obtained with confocal microscopy revealed that HDMVECs display primarily peripheral membrane localized p-FAK, indicating sites of cellular attachment to the extracellular matrix (ECM) (Figure 1C). In contrast, p-FAK localization in HEMECs was observed along the entirety of the actin stress fibers, suggesting cellular adhesion to its substrate is markedly altered in HEMECs. Indeed, it has previously been reported that HEMECs display unique expression of genes involved in cellular adhesion [8].

### Whole genome microarray analysis reveals large scale alterations in gene expression between HEMECs and HDMVECs

Given the phenotypic differences observed between HEMECs and HDMVECs, we compared the global gene expression patterns between pure cultures of these cells using Illumina high density BeadArrays to elucidate which molecular factors are deregulated in HEMECs. Our array analysis identified 125 genes whose expression was upregulated and 104 genes whose expression was downregulated (2 fold or greater, p<0.05) in HEMECs compared to HDMVECs (Table 1). Metacore analysis of the 2 fold or greater gene expression changes revealed three major classifications of gene functions that are altered in HEMECs including cell adhesion (*TIMP1, COL1A1, COL1A2, MMP1, MMP13, SERPINE2, COL4A6, LAMC2, MMP2, CD44, CAV1, CCL2, JAM3, CLDN11, LYVE1*), cell cycle (*CCND2, CDKN2A, CCNA1, NCAPD2*), and arachidonic acid production (*ACSL5, FAP, LIPG, PLA2G4C*). Given the number of adhesion genes whose expression is altered in HEMECs compared to HDMVECs, it is no surprise that we observed altered subcellular localization of p-FAK in HEMECs (Figure 1C), reflecting a unique adhesive phenotype in these cells. Our data reflect altered cell cycle regulation in HEMECs, with a downregulation of *CCND2 (*cyclin D2) and CDKN2A (p16Ink4A) and a potent 6.6 fold increase in *CCNA1* (cyclin A1), and these changes may contribute to the enhanced proliferation rates in HEMECs and the uncontrolled cell growth observed in infantile hemangiomas tumors. Alterations in the expression of genes involved in arachidonic acid production were unique in that this polyunsaturated fatty acid can serve as a lipid second messenger in the regulation of phospholipase-C and protein kinase-C signaling, is a key inflammatory intermediate, and can act as a vasodilator [9].

**Table 1:** Fold changes in mRNA expression levels of genes in HEMECs compared to HDMVECs

**Gene symbol**	**Gene name**	**Accession number**	**FC**
CTAG2	Cancer/testis antigen 2	NM_020994.3	11.6
IL13RA2	Interleukin 13 Receptor, alpha 2	NM_000640.2	10.7
IFI27	Interferon, alpha-inducible protein 27	NM_005532.3	8.3
TPM2	Tropomyosin 2 (beta)	NM_213674.1	7.8
RPL14	Ribosomal protein L14	NM_001034996.1	6.6
CCNA1	Cyclin A1	NM_003914.3	6.6
RGS5	G-protein signaling 5 regulator	NM_003617.3	6.0
FBN2	Fibrillin 2	NM_001999.3	5.9
D4S234E	DNA segment on chromosome 4 (unique)	NM_001040101.1	5.5
BST2	Bone marrow stromal cell antigen 2	NM_004335.2	5.1
QPCT	Glutaminyl-peptide cyclotransferase	NM_012413.3	4.8
TNFSF4	Tumor necrosis factor (ligand) superfamily, member 4	NM_003326.3	4.6
RGS5	Regulator of G-protein signaling 5	NM_003617.3	4.6
SPOCK1	Sparc/osteonectin, cwcv and kazal-like domains proteoglycan 1	NM_004598.3	4.6
SNHG8	Small nucleolar RNA host gene 8 (non-protein coding)	NR_003584.3	4.6
ANTXR1	Anthrax toxin receptor 1	NM_032208.2	4.5
CHST1	Carbohydrate sulfotransferase 1	NM_003654.5	4.5
MPZL2	Myelin protein zero-like 2	NM_005797.3	4.4
HEY2	Hairy/enhancer-of-spilt related with YRPW motif 2	NM_012259.2	4.3
SLITRK4	SLIT and NTRK-like family, member 4	NM_173078.3	4.2
SHISA2	Shisa homolog 2	NM_001007538.1	4.0
LRRC17	Leucine rich repeat containing 17, TV2	NM_005824.2	3.9
NUDT11	Nudix-type motif 11	NM_018159.3	3.8
RNASE1	Ribonuclease, Rnase A family, 1, TV1	NM_198235.2	3.7
SERPINE2	Serpin peptidase inhibitor, clade E, member 2	NM_006216.3	3.6
LIPG	Lipase, endothelial	NM_006033.2	3.4
PCSK5	Proprotein convertase subtilisin/kexin type 5	NM_006200.3	3.4
LPXN	Leupaxin	NM_004811.2	3.3
CXCR4	Chmeokine (C-X-C motif) receptor 4, TV2	NM_003467.2	3.2
TMEM200A	Transmembrane protein 200A	NM_052913.2	3.1
CXCR4	Chemokine (C-X-C motif) receptor 4, TV1	NM_001008540.1	3.1
RAB34	RAB34, member RAS onogene family	NM_031934.5	3.0
DPYSL3	Dihydropyrimidinase-like 3	NM_001387.2	2.9
FBXL13	F-box and leucine-rich repeat protein 13	NM_145032.3	2.9
PNMA2	Paraneoplastic Ma antigen 2	NM_007257.5	2.9
LOC440354	LOC440354	NR_002473.2	2.9
NLGN1	Neuroligin 1	NM_014932.2	2.8
DDIT4	DNA-damage-inducible transcript 4	NM_019058.2	2.8
PFN2	Profilin 2	NM_053024.3	2.8
GABBR2	Gamma-aminobutyric acid B receptor, 2	NM_005458.7	2.8
MEIS2	Meis homeobox 2	NM_172315.2	2.7
PMEPA1	Prostate transmembrane protein, androgen induced 1	NM_199169.2	2.7
LOC647307	LOC647308	XR_039752.1	2.7
PLEK2	Pleckstrin 2	NM_016445.1	2.7
CARD11	Caspase recruitment domain family, member 11	NM_032415.4	2.6
SNORD13	Small nucleolar RNA, C/D box 13, small nucleolar RNA	NR_003041.1	2.6
GFPT2	Glutamine-fructoce-6-phosphate transaminase 2	NM_005110.2	2.6
FAP	Fibroblast activation protein, alpha	NM_004460.2	2.6
OCIAD2	OCIA domain containing 2, TV2	NM_152398.2	2.5
F2RL1	Coagulation factor II receptor-like 1	NM_005242.4	2.5
DSTYK	Dual serine/threonine and tyrosine protein kinase	NM_199462.2	2.5
LOC649497	LOC649498	XM_938576.1	2.5
LOC654194	LOC654195	XM_942669.1	2.5
NYNRIN	NYN domain and retroviral integrase containing	NM_025081.2	2.5
LOC387763	LOC387764	XM_941665.2	2.5
COL8A1	Collagen, type VIII, alpha 1	NM_020351.3	2.5
MGC39900	MGC39901	XM_936687.1	2.4
LTBP2	Latent transforming growth factor beta binding protein 2	NM_000428.2	2.4
RNASE1	Ribonuclease, Rnase A family, 1, TV3	NM_198232.2	2.4
IFI27L2	Interferon, alpha-inducible protein 27-like 2	NM_032036.2	2.4
SOX4	SRY (sex determining region Y)-box4	NM_003107.2	2.4
LRRC17	Leucine rich repeat containing 17, TV1	NM_001031692.2	2.3
DSE	Dermatan sulfate epimerase	NM_013352.2	2.3
CD44	CD44 molecule (Indian blood group), TV5	NM_001001392.1	2.3
LOC100131139	LOC100131140	XR_037336.1	2.3
CBS	Systathionine-beta-synthase	NM_000071.2	2.3
NT5DC2	5'-nucleotidase domain containing 2	NM_022908.2	2.3
NPFFR2	Neuropeptide FF receptor 2	NM_004885.2	2.3
LOC100129685	LOC100129686	XM_001723814.1	2.3
LXN	Latexin	NM_020169.3	2.3
MEX3B	Mex-3 homolog B	NM_032246.3	2.3
C1orf54	Chromosome 1 open reading frame 54	NM_024579.3	2.3
HDDC2	HD domain containing 2	NM_016063.2	2.3
LOC648823	LOC648824	XM_943477.1	2.3
CYB5A	Cytochrome b5 type A	NM_001914.3	2.3
PIR	Pirin (iron binding nuclear protein)	NM_001018109.2	2.3
GPR37	G protein-coupled receptor 37	NM_005302.2	2.3
PPAPDC1A	Phosphatidic acid phosphatase type 2 domain containing 1A	NM_001030059.1	2.3
CD44	CD44 molecule (Indian blood group), TV4	NM_001001391.1	2.2
LOC100131905	LOC100131906	XR_039334.1	2.2
CTAG1A	Cancer/testis antigen 1A	NM_139250.1	2.2
C4orf18	Chromosome 4 open reading frame 18	NM_016613.6	2.2
LDOC1	Leucine zipper, down-regulated in cancer 1	NM_012317.2	2.2
TGFBI	Transforming growth factor, beta-induced	NM_000358.2	2.2
COL5A2	Collagen, type V, alpha 2	NM_000393.3	2.2
NOX4	NADPH oxidase 4	NM_016931.3	2.2
TSHZ3	Teashirt zinc finger homeobox 3	NM_020856.2	2.2
FNDC3B	Fibronectin type III domain containing 3B, TV2	NM_001135095.1	2.2
KIT	V-kit	NM_001093772.1	2.2
ADAM19	ADAM metallopeptidase domain 19	NM_033274.3	2.2
JAM3	Junctional adhesion molecule 3	NM_032801.4	2.1
CGNL1	Cingulin-like 1	NM_032866.4	2.1
COL4A6	Collagen, type IV, alpha 6	NM_001847.2	2.1
BMX	BMX non-receptor tyrosine kinase	NM_001721.6	2.1
DUSP23	Dual specificity phosphatase 23	NM_017823.3	2.1
MMP2	Matrix metallopeptidase 2	NM_004530.4	2.1
NCAPD2	Non-SMC condensin I complex, subunit D2	NM_014865.3	2.1
CYBRD1	Cytochrome b reductase 1, TV1	NM_024843.2	2.1
FAM89A	Family with sequence similarity 89, member A	NM_198552.2	2.1
GAS6	Growth arrest-specific 6	NM_000820.2	2.1
S100A13	S100 calcium binding protein A13	NM_001024211.1	2.1
SMARCE1	SWI/SNF related, subfamily e, member 1	NM_003079.4	2.1
LOC643977	LOC643978	XM_932991.1	2.1
LFNG	O-fucosylpeptide 3-beta-N-acetylglucosaminyltransferase	NM_001040167.1	2.1
MTMR11	Myotubularin related protein 11	NM_181873.3	2.1
ITGA10	Integrin, alpha 10	NM_003637.3	2.1
PTGFRN	Prostaglandin F2 receptor negative regulator	NM_020440.2	2.0
LOC644936	Actin, beta pseudogene	NR_004845.1	2.0
CPS1	Carbamoyl-phosphate synthase 1, mitochonfrial	NM_001875.4	2.0
C18orf56	Chromosome 18 open reading frame 56	NM_001012716.2	2.0
ADA	Adenosine deaminase	NM_000022.2	2.0
NETO2	Neuropilin and tolliod-like2	NM_018092.4	2.0
DKFZp761P0423	DKFZp761P0424	XM_291277.4	2.0
STC2	Stanniocalcin 2	NM_003714.2	2.0
PRKAR1A	Protein kinase, cAMP-dependent, regulatory, type I, alpha	NM_002734.3	2.0
EGFLAM	EGF-like, fibronectin type III and laminin G domains	NM_182801.2	2.0
SPECC1	Sperm antigen with calponin homology, coiled-coil domains 1	NM_001033555.2	2.0
FNDC3B	Fibronectin type III domain containing 3B, TV1	NM_022763.3	2.0
THOC3	THO complex 3	NM_032361.2	2.0
COL5A1	Collagen, type V, alpha 1	NM_000093.3	2.0
LANCL1	LanC lantibiotic synthetase component C-like 1	NM_006055.2	2.0
OCIAD2	OCIA domain containing 2, TV1	NM_001014446.1	2.0
LRIG1	Leucine-rich repeats and immunoglobulin-like domains 1	NM_015541.2	2.0
HOXB2	Homeobox B2	NM_002145.3	2.0
TIMP1	TIMP metallopeptidase inhibitor 1	NM_003254.2	−2.0
NAAA	N-acylethanolamine acid amidase	NM_014435.3	−2.0
MAOA	Monoamine oxidase A	NM_000240.2	−2.0
MYOF	Myoferlin	NM_013451.3	−2.0
KISS1	KiSS metastasis-suppressor	NM_002256.3	−2.0
SLC25A22	Solute carrier family 25, member 22	NM_024698.5	−2.0
NOSIP	Nitric oxide synthase interacting protein	NM_015953.3	−2.0
COL1A2	Collagen, type I, alpha 2	NM_000089.3	−2.0
ZDHHC14	Zinc finger, DHHC-type containing 14	NM_024630.2	−2.0
HPCAL1	Hippocalcin-like 1	NM_134421.1	−2.0
VLDLR	Very low density lipoprotein receptor	NM_001018056.1	−2.0
LOC730525	LOC730525	XM_001126202.1	−2.0
BMP2	Bone morphogenetic protein 2	NM_001200.2	−2.0
ABLIM1	Actin binding LIM protein 1	NM_006720.3	−2.0
PIK3C2A	Phosphoinositide-3-kinase, class 2, alpha polypeptide	NM_002645.2	−2.0
IRF1	Interferon regulatory factor 1	NM_002198.2	−2.0
MBP	Myelin basic protein	NM_001025100.1	−2.0
PRKAR1B	Protein kinase, cAMP-dependent, regulatory type I, beta	NM_002735.2	−2.1
FAM101B	Family with sequence similarity 101, member B	NM_182705.2	−2.1
ERCC2	DNA excision repair protein 2	NM_000400.3	−2.1
CCND2	Cyclin D2	NM_001759.3	−2.1
HLA-B	Major histocompatibility complex, class I, B	NM_005514.6	−2.1
SYBU	Syntabulin	NM_001099743.1	−2.1
PDE2A	Phosphodiesterase 2A, cGMP-stimulated	NM_002599.4	−2.1
AKAP12	A kinase anchor protein 12	NM_005100.3	−2.1
CLEC2B	C-type lectin domain family 2, member B	NM_005127.2	−2.1
S100A4	S100 calcuim binding protein A4	NM_019554.2	−2.1
FST	Follistain	NM_013409.2	−2.2
SLC30A3	Solute carrier family 30, member 3	NM_003459.4	−2.2
PLIN2	Perilipin 2	NM_001122.3	−2.2
IL32	Interleukin 32	NM_001012633.1	−2.2
LOC100128252	LOC100128253	XM_001725603.1	−2.2
TIMM22	Translocase of inner mitochondrial membrane 22 homolog	NM_013337.2	−2.2
SYNM	Synemin, intermediate filament protein	NM_015286.5	−2.2
LOC729985	LOC729986	XM_001131964.1	−2.2
ADRB2	Adrenergic, beta-2-, receptor surface	NM_000024.5	−2.2
KIAA1274	KIAA1274	NM_014431.2	−2.2
PRR5	Proline rich 5	NM_001017529.2	−2.2
LOC387841	LOC387842	XM_932678.1	−2.3
CFI	Complement factor I	NM_000204.3	−2.3
LOC646836	LOC646837	XM_001718162.1	−2.3
COL1A1	Collagen, type I, alpha 1	NM_000088.3	−2.3
CCL2	Chemokine (C-C motif) ligand 2	NM_002982.3	−2.3
COL6A1	Collagen, type VI, alpha 1	NM_001848.2	−2.3
LOC201651	LOC201652	XR_017321.2	−2.3
GALNTL4	GalNAc-T-like protein 4	NM_198516.2	−2.3
S100A3	S100 calcuim binding protein A3	NM_002960.1	−2.4
ALDH1A1	Aldehyde dehydrogenase 1 family, member A1	NM_000689.4	−2.4
TNFRSF14	Tumor necosis factor receptor superfamily, member 14	NM_003820.2	−2.4
CAV1	Caveolin 1	NM_001753.4	−2.4
LAMC2	Laminin, gamma 2	NM_005562.2	−2.4
NOSTRIN	Nitric oxide synthase trafficker	NM_052946.3	−2.4
CEACAM1	Carcinoembryonic antigen-related cell adhesion molecule 1	NM_001024912.2	−2.4
CYYR1	Cysteine/tyrosine-rich 1	NM_052954.2	−2.5
SLC22A23	Solute carrier family 22, member 23	NM_021945.5	−2.5
ACSL5	Acyl-CoA synthetase long-chain family member 5	NM_016234.3	−2.5
AADAC	Arylacetamide deacetylase	NM_001086.2	−2.6
COLEC12	Collectin sub-family member 12	NM_130386.2	−2.6
KIAA1324L	KIAA1324-like	NM_152748.3	−2.6
RNASET2	Ribonuclease T2	NM_003730.4	−2.6
NXN	Nucleoredoxin	NM_022463.4	−2.6
PLA2G4C	Phospholipase A2, group IVC	NM_003706.2	−2.6
SERPINB2	Serpin peptidase inhibitor, clade B, member 2	NM_002575.2	−2.6
CETP	Cholesteryl ester transfer protein, plasma	NM_000078.2	−2.7
PLA2G16	Phospholipase A2, group XVI	NM_007069.3	−2.7
TNFSF18	Tumor necrosis factor superfamily, member 18	NM_005092.3	−2.8
CITED2	Cbp/p300-interacting transactivator 2	NM_006079.3	−2.8
C10orf116	Chromosome 10 open reading fame 116	NM_006829.2	−2.8
PROX1	Prospero homeobox 1	NM_002763.3	−2.9
PALM	Paralemmin	NM_002579.2	−2.9
ZSCAN18	Zinc finger and SCAN domain containing 18	NM_023926.4	−2.9
LEPREL1	Leprecan-like 1	NM_018192.3	−2.9
CTSH	Cathepsin H	NM_004390.3	−2.9
KHDRBS3	RNA-binding protein T-Star	NM_006558.1	−3.0
CDH11	Cadherin 11, type 2, OB-cadherin	NM_001797.2	−3.1
DDIT4L	DNA-damage-inducible transcript 4-like	NM_145244.3	−3.2
GAPDHL6	GAPDHL7	XM_001726954.1	−3.2
NR5A2	Nuclear receptor subfamily 5, group A, member 2	NM_003822.3	−3.3
ABCA3	ATP-binding cassette, sub-family A, member 3	NM_001089.2	−3.3
MARCH2	Membrane-associated ring finger 2	NM_001005416.1	−3.3
CDKN2A	Cyclin-dependent kinase inhibitor 2A	NM_000077.4	−3.3
MGP	Matrix Gla protein	NM_000900.3	−3.3
ALDH1A2	Aldehyde dehydrogenase 1 family, member A2	NM_170697.2	−3.5
HOXB7	Homeobox B7	NM_004502.3	−3.5
EMCN	Endomucin	NM_016242.3	−3.5
ANGPT2	Angiopoietin 2	NM_001147.2	−3.5
GIMAP5	GTPase, IMAP family member 5	NM_018384.4	−3.6
NDN	Necdin homolog	NM_002487.2	−3.8
TACSTD2	Tumor associate calcuim signal transducer 2	NM_002353.2	−3.8
KRT19	Keratin 19	NM_002276.4	−3.8
FAM174B	Family with sequence similarity 174, member B	NM_207446.2	−3.9
CECR1	Cat eye syndrome chromosome region, candidate 1	NM_177405.1	−4.2
GPR116	G protein-coupled receptor 116	NM_015234.4	−4.3
TNFRSF6B	Tumor necrosis factor superfamily, member 6b, decoy	NM_032945.2	−4.3
PIEZO2	Piezo-type mechanosensitive ion channel component 2	NM_022068.2	−4.4
UCHL1	Ubiquitin carboxyl-terminal esterase L1	NM_004181.4	−4.9
KBTBD11	Kelch repeat and BTB domain containing 11	NM_014867.2	−5.3
LOC375295	LOC375296	XM_374020.4	−5.5
HSD17B2	Hydroxysteroid dehydrogenase 2	NM_002153.2	−8.4
LYVE1	Lymphatic vessel endothelial hyaluronan receptor 1	NM_006691.3	−8.8
PDPN	Podoplanin	NM_001006625.1	−15.8
GYPC	Glycophorin C	NM_016815.3	−22.6
MMP1	Matrix metallopeptidase 1	NM_002421.3	−25.8
FABP4	Fatty acid binding protein 4, adipocyte	NM_001442.2	−28.1
CLDN11	Claudin 11	NM_005602.5	−36.9

We confirmed a small subset of these gene expression changes utilizing qPCR, revealing equivocal trends in gene expression between the microarray and qPCR data for *ANGPT2, ANTXR1, SMARCE1, RGS5, CTAG2, LTBP2, CLDN11,* and *KISS1* (Table 2). Each of these genes has been firmly established to play critical roles in regulating angiogenesis and/or tumor progression [10-17]. Missense mutations in *ANTXR1* have been reported in several infantile hemangiomas and contribute to the constitutive VEGFR2 signaling associated with these tumors [18]. Mutations and signaling aberrations in Tie2, the cognate receptor for ANGPT2, play central roles in the development of various vascular disorders [19,20]. ANGPT2 has previously been shown to be down-regulated in response to serum in HEMECs [19]. Interestingly, ANGPT2 expression is higher in HEMECs compared to normal placental endothelial cells and is increased in proliferative infantile hemangioma tumors relative to involuting ones [5]. Virtually undetectable in normal vasculature, *RGS5* is greatly upregulated in the vasculature of solid tumors and may have the potential to serve as a tumor biomarker [12]. The downregulation of the metastasis suppressor *KISS1* that we observed in HEMECs may partially explain the locally aggressive properties of infantile hemangiomas, as this gene encodes an angiogenic suppressor [16,21]. Moreover, the expression of *KISS1* is markedly reduced in aggressive metastatic melanomas and breast cancers, and this loss of expression contributes to the metastatic phenotype of these cells [17,22]. It is intriguing that such genes (particularly the cancer-specific genes) are aberrantly expressed in HEMECs, and undoubtedly their deregulation could potentiate aberrant vascular tumor states. As it has been proposed that infantile hemangiomas may be derived from motile placental-derived chorangioma cells [2], future genomics analysis should compare the transcriptomes of each tumor type to identify if aberrant expression of tumor-related genes is shared between the tissues.

**Table 2:** qPCR confirmation of a subset of gene expression changes in HEMECs compared to HDMVECs

**Gene**	**Expression Δ**
RGS5	92.4 ± 11.2
CTAG2	39.9 ± 4.8
SMARCE1	4.4 ± 1.4
LTBP2	3.3 ± 0.5
ANGPT2	−2.1 ± 0.3
KISS1	−2.5 ± 0.4
ANTXR1	−2.8 ± 0.4
CLDN11	−10.0 ± 0.9

p≤0.05 for all values.

### Overexpression of the CTAG2 cancer/testis antigen in a panel of infantile hemangioma tumors

**Figure 2:**
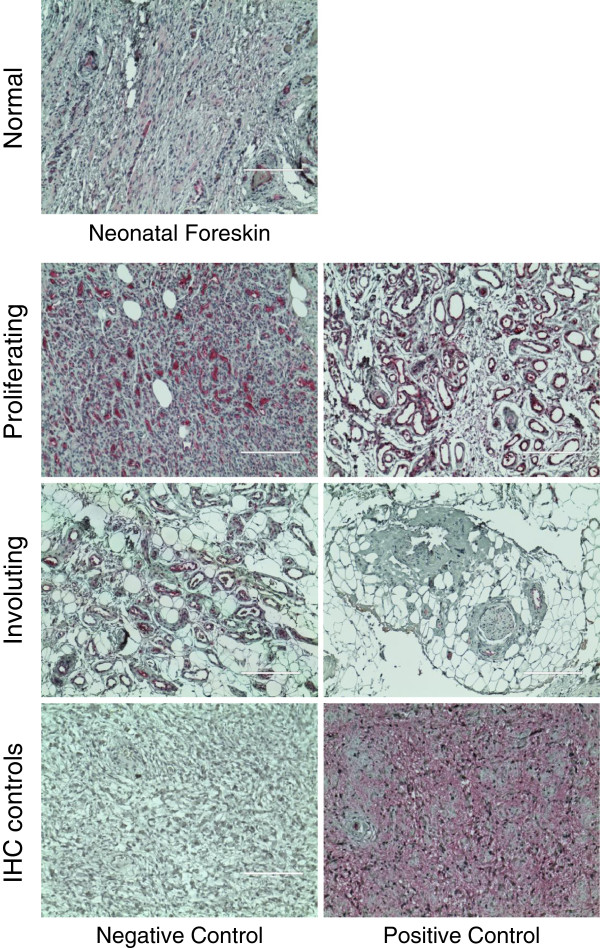
**Detection of CTAG2 protein levels in infantile hemangioma tissues.** Proliferating and involuting infantile hemangioma tissues as well as normal neonatal foreskin tissues were cut from paraffin blocks, incubated with antibodies against CTAG2, and detected using alkaline phosphatase staining (*red*). Immunohistochemistry (*IHC*) controls included incubations without CTAG2 antibody (*negative control*) and with CTAG2 antibody (*positive control*) in thin sections from metastatic breast cancer. All images were obtained at 100X total magnification.

In our microarray analysis, the cancer/testis antigen CTAG2 displayed the highest upregulation of mRNA expression in HEMECs compared to the HDMECs. This gene, whose function is completely unknown, has been shown to be significantly increased in several metastatic cancers, and is actively being researched as a target of immune therapy for aggressive cancers [23-29]. If CTAG2 is preferentially upregulated in infantile hemangiomas, it is possible that treatment of disfiguring or life threatening infantile hemangioma tumors could employ immune therapy against this antigen. Furthermore, CTAG2 is reported to be a target for antigen-specific T-cells in patients with various metastatic tumors [29,30]. A recent study has shown that nearly half of the patients with spontaneous CTAG2-specific CD4(+) T cell responses had circulating CTAG2-specific antibodies that recognized epitopes located in the C-terminal portion of CTAG2 [30]. As involution of infantile hemangiomas is believed to be due in part to an immune mediated attack on the tumor itself [4], it is possible that T-cell targeting of the overexpressed CTAG2 protein could contribute to this process. We confirmed our microarray data at the protein level by performing immunohistochemistry on a panel of 16 paraffin embedded infantile hemangioma tumors representing both the proliferating and involuting stages of the disease and 4 normal neonatal dermal tissues. A limited amount of CTAG2 expression was observed in the normal dermal tissues (a few nerve cells and bundles present staining, whereas the fibroblasts and collagen fibers are negative), and despite this gene being coined a “cancer/testis specific antigen”, analysis of publically available microarray datasets suggests this gene is expressed at a low level across a large number of tissues (http://www.biogps.org) and it has been reported in the literature to be expressed in the placenta and ovary [31]. In proliferating tumors (composed of densely proliferating endothelial cells), we observed intense CTAG2 staining in the endothelial cells for all sections analyzed (Figure 2). In contrast, involuting tumors (marked by substantial adipocyte deposits—a characteristic of the later stages in the development of this tumor [32]) exhibited significantly reduced levels of CTAG2 staining. As Calicchio et al. did not detect significant differences in CTAG2 expression between microdissected endothelial cells from proliferating and involuting infantile hemangiomas and the staining intensity of individual blood vessels appears relatively constant between proliferating and involuting hemangiomas, we suspect that the reduced CTAG2 staining in involuting tumors is most likely due to reductions in tumor vascular density but not changes in gene transcription.

## Conclusion

Our data indicate that global transcriptional expression patterns are markedly unique between pure cultures of HDMVECs and HEMECs with major alterations in cell cycle, adhesion, and arachidonic acid metabolism genes. Though considered benign, HEMECs showed surprising aberrant regulation in the expression of several genes involved in tumor progression. Our finding that CTAG2 is highly expressed in infantile hemangiomas may lead to the development of immune-mediated therapies against infantile hemangiomas.

## Competing interests

The authors declare that they have no competing interests.

## Authors’ contributions

JMS Performed proliferation and migration assays, confocal microscopy, and qPCR RKR Cultured the HDMVEC and HIHECs and prepared samples for microarray analysis. CA Generated and analyzed tables of statistically significant genes, performed bioinformatics analysis to identify key gene networks. DD Performed histology and immunohistochemistry. VK Provided human tissues and expertise in histological and immunohistochemistry analysis. DCM Performed microarray analysis and bioinformatics analysisc. BAB Wrote manuscript, analyzed data, coordinated project.

## References

[B1] North PE Waner M Mizeracki A Mihm MC Jr GLUT1: a newly discovered immunohistochemical marker for juvenile hemangiomas Hum Pathol 2000 31 11 22 10.1016/S0046-8177(00)80192-6 10665907

[B2] Mihm MC Jr Nelson JS Hypothesis: the metastatic niche theory can elucidate infantile hemangioma development J Cutan Pathol 2010 37 Suppl 1 83 87 20482680 10.1111/j.1600-0560.2010.01521.x PMC3177757

[B3] Barnes CM Huang S Kaipainen A Sanoudou D Chen EJ Eichler GS Guo Y Yu Y Ingber DE Mulliken JB Beggs AH Folkman J Fishman SJ Evidence by molecular profiling for a placental origin of infantile hemangioma Proc Natl Acad Sci USA 2005 102 19097 19102 10.1073/pnas.0509579102 16365311 PMC1323205

[B4] Ritter MR Moreno SK Dorrell MI Rubens J Ney J Friedlander DF Bergman J Cunningham BB Eichenfield L Reinisch J Cohen S Veccione T Holmes R Friedlander SF Friedlander M Identifying potential regulators of infantile hemangioma progression through large-scale expression analysis: a possible role for the immune system and indoleamine 2,3 dioxygenase (IDO) during involution Lymphat Res Biol 2003 1 291 299 10.1089/153968503322758094 15624557

[B5] Calicchio ML Collins T Kozakewich HP Identification of signaling systems in proliferating and involuting phase infantile hemangiomas by genome-wide transcriptional profiling Am J Pathol 2009 174 1638 1649 10.2353/ajpath.2009.080517 19349369 PMC2671253

[B6] Boye E Yu Y Paranya G Mulliken JB Olsen BR Bischoff J Clonality and altered behavior of endothelial cells from hemangiomas J Clin Invest 2001 107 745 752 10.1172/JCI11432 11254674 PMC208946

[B7] Stiles J Amaya C Pham R Rowntree RK Lacaze M Mulne A Bischoff J Kokta V Boucheron LE Mitchell DC Bryan BA Propranolol treatment of infantile hemangioma endothelial cells: A molecular analysis Exp Ther Med 2012 4 594 604 23170111 10.3892/etm.2012.654 PMC3501380

[B8] Khan ZA Melero-Martin JM Wu X Paruchuri S Boscolo E Mulliken JB Bischoff J Endothelial progenitor cells from infantile hemangioma and umbilical cord blood display unique cellular responses to endostatin Blood 2006 108 915 921 10.1182/blood-2006-03-006478 16861344 PMC1895853

[B9] Pfister SL Gauthier KM Campbell WB Vascular pharmacology of epoxyeicosatrienoic acids Adv Pharmacol 2010 60 27 59 21081214 10.1016/B978-0-12-385061-4.00002-7 PMC3373307

[B10] Fagiani E Christofori G Angiopoietins in angiogenesis Cancer Lett 2013 328 18 26 10.1016/j.canlet.2012.08.018 22922303

[B11] Chaudhary A St Croix B Selective blockade of tumor angiogenesis Cell Cycle 2012 11 2253 2259 10.4161/cc.20374 22617387 PMC3383587

[B12] Silini A Ghilardi C Figini S Sangalli F Fruscio R Dahse R Pedley RB Giavazzi R Bani M Regulator of G-protein signaling 5 (RGS5) protein: a novel marker of cancer vasculature elicited and sustained by the tumor's proangiogenic microenvironment Cell Mol Life Sci 2012 69 1167 1178 10.1007/s00018-011-0862-8 22130514 PMC3299962

[B13] Garcia-Pedrero JM Kiskinis E Parker MG Belandia B The SWI/SNF chromatin remodeling subunit BAF57 is a critical regulator of estrogen receptor function in breast cancer cells J Biol Chem 2006 281 22656 22664 10.1074/jbc.M602561200 16769725

[B14] Lethe B Lucas S Michaux L De Smet C Godelaine D Serrano A De Plaen E Boon T LAGE-1, a new gene with tumor specificity Int J Cancer 1998 76 903 908 10.1002/(SICI)1097-0215(19980610)76:6<903::AID-IJC22>3.0.CO;2-1 9626360

[B15] Wessells H Sullivan CJ Tsubota Y Engel KL Kim B Olson NE Thorner D Chitaley K Transcriptional profiling of human cavernosal endothelial cells reveals distinctive cell adhesion phenotype and role for claudin 11 in vascular barrier function Physiol Genomics 2009 39 100 108 10.1152/physiolgenomics.90354.2008 19622796 PMC2765067

[B16] Cho SG Yi Z Pang X Yi T Wang Y Luo J Wu Z Li D Liu M Kisspeptin-10, a KISS1-derived decapeptide, inhibits tumor angiogenesis by suppressing Sp1-mediated VEGF expression and FAK/Rho GTPase activation Cancer Res 2009 69 7062 7070 10.1158/0008-5472.CAN-09-0476 19671799 PMC3242001

[B17] Mitchell DC Stafford LJ Li D Bar-Eli M Liu M Transcriptional regulation of KiSS-1 gene expression in metastatic melanoma by specificity protein-1 and its coactivator DRIP-130 Oncogene 2007 26 1739 1747 10.1038/sj.onc.1209963 16964286

[B18] Jinnin M Medici D Park L Limaye N Liu Y Boscolo E Bischoff J Vikkula M Boye E Olsen BR Suppressed NFAT-dependent VEGFR1 expression and constitutive VEGFR2 signaling in infantile hemangioma Nat Med 2008 14 1236 1246 10.1038/nm.1877 18931684 PMC2593632

[B19] Yu Y Varughese J Brown LF Mulliken JB Bischoff J Increased Tie2 expression, enhanced response to angiopoietin-1, and dysregulated angiopoietin-2 expression in hemangioma-derived endothelial cells Am J Pathol 2001 159 2271 2280 10.1016/S0002-9440(10)63077-5 11733376 PMC1850579

[B20] London NR Whitehead KJ Li DY Endogenous endothelial cell signaling systems maintain vascular stability Angiogenesis 2009 12 149 158 10.1007/s10456-009-9130-z 19172407 PMC2698036

[B21] Ramaesh T Logie JJ Roseweir AK Millar RP Walker BR Hadoke PW Reynolds RM Kisspeptin-10 inhibits angiogenesis in human placental vessels ex vivo and endothelial cells in vitro Endocrinology 2010 151 5927 5934 10.1210/en.2010-0565 20926586

[B22] Mitchell DC Abdelrahim M Weng J Stafford LJ Safe S Bar-Eli M Liu M Regulation of KiSS-1 metastasis suppressor gene expression in breast cancer cells by direct interaction of transcription factors activator protein-2alpha and specificity protein-1 J Biol Chem 2006 281 51 58 16260418 10.1074/jbc.M506245200

[B23] Odunsi K Jungbluth AA Stockert E Qian F Gnjatic S Tammela J Intengan M Beck A Keitz B Santiago D Williamson B Scanlan MJ Ritter G Chen YT Driscoll D Sood A Lele S Old LJ NY-ESO-1 and LAGE-1 cancer-testis antigens are potential targets for immunotherapy in epithelial ovarian cancer Cancer Res 2003 63 6076 6083 14522938

[B24] Zeng G Aldridge ME Wang Y Pantuck AJ Wang AY Liu YX Han Y Yuan YH Robbins PF Dubinett SM deKernion JB Belldegrun AS Dominant B cell epitope from NY-ESO-1 recognized by sera from a wide spectrum of cancer patients: implications as a potential biomarker Int J Cancer 2005 114 268 273 10.1002/ijc.20716 15540228

[B25] Kan T Yamasaki S Kondo K Teratani N Kawabe A Kaganoi J Meltzer SJ Imamura M Shimada Y A new specific gene expression in squamous cell carcinoma of the esophagus detected using representational difference analysis and cDNA microarray Oncology 2006 70 25 33 10.1159/000091183 16446548

[B26] Shao Y Sun ZY Sun SW Zhao Y Sin WY Yuan YH Simpson AJ Old LJ Sang XT Mao YL Xie Y Huang JF Zhao HT Identification and expression analysis of novel LAGE-1 alleles with single nucleotide polymorphisms in cancer patients J Cancer Res Clin Oncol 2008 134 495 502 10.1007/s00432-007-0312-z 17899192 PMC12161627

[B27] Andrade VC Vettore AL Felix RS Almeida MS Carvalho F Oliveira JS Chauffaille ML Andriolo A Caballero OL Zago MA Colleoni GW Prognostic impact of cancer/testis antigen expression in advanced stage multiple myeloma patients Cancer Immun 2008 8 2 18237105 PMC2935785

[B28] Wang XY Chen HS Luo S Zhang HH Fei R Cai J Comparisons for detecting NY-ESO-1 mRNA expression levels in hepatocellular carcinoma tissues Oncol Rep 2009 21 713 719 19212631

[B29] Pollack SM Li Y Blaisdell MJ Farrar EA Chou J Hoch BL Loggers ET Rodler E Eary JF Conrad EU 3rd Jones RL Yee C NYESO-1/LAGE-1s and PRAME are targets for antigen specific T cells in chondrosarcoma following treatment with 5-Aza-2-deoxycitabine PLoS One 2012 7 e32165 10.1371/journal.pone.0032165 22384167 PMC3288075

[B30] Kudela P Sun Z Fourcade J Janjic B Kirkwood JM Maillere B Zarour HM Epitope hierarchy of spontaneous CD4+ T cell responses to LAGE-1 J Immunol 2011 186 312 322 10.4049/jimmunol.1001989 21131422 PMC3901358

[B31] Kalejs M Erenpreisa J Cancer/testis antigens and gametogenesis: a review and "brain-storming" session Cancer Cell Int 2005 5 4 10.1186/1475-2867-5-4 15715909 PMC552320

[B32] Kleiman A Keats EC Chan NG Khan ZA Evolution of hemangioma endothelium Exp Mol Pathol 2012 93 264 272 10.1016/j.yexmp.2012.04.020 22565125

